# Sex and Biology: Broader Impacts Beyond the Binary

**DOI:** 10.1093/icb/icad113

**Published:** 2023-08-17

**Authors:** Sam L Sharpe, Andrew P Anderson, Idelle Cooper, Timothy Y James, Alexandra E Kralick, Hans Lindahl, Sara E Lipshutz, J F McLaughlin, Banu Subramaniam, Alicia Roth Weigel, A Kelsey Lewis

**Affiliations:** Division of Biology, Kansas State University, Manhattan, KS 66506, USA; Department of Biology, Reed College, Portland, OR 97202, USA; Department of Biology, James Madison University, Harrisonburg, VA 22807, USA; Department of Ecology and Evolutionary Biology, University of Michigan, Ann Arbor, MI 48109, USA; Department of Anthropology, University of Pennsylvania, Philadelphia, PA 19104, USA; Independent writer and activist; Department of Biology, Loyola University Chicago, Chicago, IL 60660, USA; Department of Biology, Duke University, Durham, NC 27708, USA; Department of Environmental Science, Policy, and Management, University of California Berkeley, Berkeley, CA 94720, USA; Department of Women, Gender, Sexuality Studies, University of Massachusetts-Amherst, Amherst, MA 01003, USA; Austin Human Rights Commissioner and activist; Department of Global Gender and Sexuality Studies, University at Buffalo-SUNY, Buffalo, New York 14260, USA

## Abstract

What are the implications of misunderstanding sex as a binary, and why is it essential for scientists to incorporate a more expansive view of biological sex in our teaching and research? This roundtable will include many of our symposium speakers, including biologists and intersex advocates, to discuss these topics and visibilize the link between ongoing reification of dyadic sex within scientific communities and the social, political, and medical oppression faced by queer, transgender, and especially intersex communities. As with the symposium as a whole, this conversation is designed to bring together empirical research and implementation of equity, inclusion, and justice principles, which are often siloed into separate rooms and conversations at academic conferences. Given the local and national attacks on the rights of intersex individuals and access to medical care and bodily autonomy, this interdisciplinary discussion is both timely and urgent.

## Introduction

In this roundtable, we discuss the present and the future of how the field of biology approaches sex, and the interplay between biology and society. We drew inspiration from a symposium presented at the Society for Integrative and Comparative Biology's 2020 Annual Conference "Reproduction: the female perspective from an integrative and comparative framework" that was co-organized by Teri Orr and Virginia Hayssen, in particular regarding this roundtable in its presentation and its summary paper (Orr et al. 2020). In line with the topic and goals of our symposium, the roundtable discussion brought together both intersex activists and biologists working in a variety of systems across taxa who are critically engaging with language and concepts surrounding biological sex. We see the collaboration of these perspectives as critical to creating strategies for implementing a broader and more accurate conceptualization of sex diversity and its implications into academic biology. Our goal is to incentivize changes in practice and perspective throughout the field, and to help develop approaches to doing so that are understood by our colleagues to be both urgent and possible. After the symposium, this article was populated with the conversations that occurred during and as a result of the following questions. This article also provides a list of recommended resources compiled by the authors (Table 1).

**Table 1:** Recommended resources.

Author(s)	Title	Publication date	Publication medium	Available from
*Biology Education Resources*				
Ah-King M	Queering animal sexual behavior in biology textbooks	2013	Confero: Queering School, Queers in School; 1(2):46–89	https://doi.org/10.3384/confero.2001-4562.13v1i21d
Barcelos CA	Transfeminist Pedagogy and the Women’s Health Classroom	2019	Feminist Formations; 31(3):1–24	https://doi.org/10.1353/ff.2019.0028
Casper AA, Rebolledo N, Lane AK, Jude L, Eddy SL	“It’s completely erasure”: A Qualitative Exploration of Experiences of Transgender, Nonbinary, Gender Nonconforming, and Questioning Students in Biology Courses	2022	CBE—Life Sciences Education; 21(4):ar69,1–19	https://doi.org/10.1187/cbe.21-12-0343
Casper AMA, Atadero RA, Fuselier LC	Revealing the queer-spectrum in STEM through robust demographic data collection in undergraduate engineering and computer science courses at four institutions	2022	PloS one; 17(3): e0264267, 1–28	https://doi.org/10.1371/journal.pone.0264267
McLaughlin J	Trans Inclusion in the Biology Classroom	2022 Feb 14	Jess McLaughlin, PhD: Blog. [Internet]	https://www.jfmclaughlin.org/blog/trans-inclusion-in-the-biology-classroom
Sharpe S	Developing LGBTQIA + Inclusive Biology Classrooms and Curricula	2022	SICB + Talk on YouTube [Internet]	https://www.youtube.com/watch?v=WA-F2cW8jZA
Zemenick AT, Turney S, Webster AJ, Jones SC, Weber MG	Six Principles for Embracing Gender and Sexual Diversity in Postsecondary Biology Classrooms	2022	BioScience; 72(5):481–492	https://doi.org/10.1093/biosci/biac013
*Biology Research Resources*				
DuBois LZ, Shattuck-Heidorn H	Challenging the binary: Gender/sex and the bio-logics of normalcy	2021	Am J Human Biology; 33(5):e23623	https://doi.org/10.1002/ajhb.23623
GenderSci Lab	Research Handout for Scientists: When and how can you apply sex contextualism in your own research?	2022 April12	GenderSci Lab: Blog [Internet]	https://www.genderscilab.org/blog/faq-for-scientists-applying-sex-contextualism
Richardson SS	Sex Contextualism	2022	Philosophy, Theory, and Practice in Biology; 14(2)	https://doi.org/10.3998/ptpbio.2096
Saguy T, Reifen-Tagar M, Joel D	The gender-binary cycle: the perpetual relations between a biological-essentialist view of gender, gender ideology, and gender-labelling and sorting	2021	Phil Trans Roy Soc B; 376(1822)	https://doi.org/10.1098/rstb.2020.0141
*Biology Perspectives*				
Ainsworth C	Sex redefined	2015	Nature; 518(7539):288–291.	https://doi.org/10.1038/518288a
Montañez A	Beyond XX and XY: The Extraordinary Complexity of Sex Determination	2017	Scientific American [Internet]; 317(3):50–51	https://doi.org/10.1038/scientificamerican0917-50
Roughgarden J	Evolution’s Rainbow: Diversity, Gender, and Sexuality in Nature and People	2013	[Book]	10th ed. Oakland (CA): University of California Press.
*Interdisciplinary Perspectives*				
Canaday M	The Straight State: Sexuality and Citizenship in Twentieth-Century America	2009	[Book]	Princeton (NJ): Princeton University Press
Costello CG	Intersex and Trans* Communities: Commonalities and Tensions; in Horlacher S. (ed). Transgender and Intersex: Theoretical, Practical, and Artistic Perspectives	2016	[Book Chapter]	New York (NY): Palgrave Macmillan. p. 83–113; https://doi.org/10.1057/978-1-349-71325-7_4
Davis G	Contesting Intersex: The Dubious Diagnosis	2015	[Book]	New York (NY): New York University Press
Karkazis K	Fixing Sex: Intersex, Medical Authority, And Lived Experience	2008	[Book]	Durham (NC): Duke University Press
Malatino H	Queer Embodiment: Monstrosity, Medical Violence, and Intersex Experience	2019	[Book]	Lincoln (NE): University of Nebraska Press
Orr C	Cripping Intersex	2022	[Book]	Vancouver (CA): University of British Columbia Press
Reis E	Bodies in Doubt: An American History of Intersex	2012	[Book]	Baltimore (MD): The Johns Hopkins University Press
Samuels EJ	Fantasies of Identification: Disability, Gender, Race	2014	[Book]	New York (NY): New York University Press.
Subramaniam B	Ghost Stories for Darwin: The Science of Variation and the Politics of Diversity	2014	[Book]	Champaign (IL): University of Illinois Press.
Viloria H, Nieto M	The Spectrum of Sex: The Science of Male, Female, and Intersex	2020	[Book]	London (UK): Jessica Kingsley Publishers
*Intersex Resources*				
Costello CG	The Phalloclitoris: Anatomy and Ideology	2011 Jan 31	The Intersex Roadshow [Internet]	https://intersexroadshow.blogspot.com/2011/01/phalloclitoris-anatomy-and-ideology.html
Costello CG	Intersex Genitalia Illustrated and Explained	2011 Apr 29	The Intersex Roadshow [Internet]	https://intersexroadshow.blogspot.com/2011/04/intersex-genitalia-illustrated-and.html
Ellis P	Category: Woman	2022	[Film]	https://www.categorywomandoc.com/
Henderson K	J.K. Rowling and the White Supremacist History of “Biological Sex.”	2020 Jul 28	Radical History Review: The Abusable Past [Internet]	www.radicalhistoryreview.org/abusablepast/j-k-rowling-and-the-white-supremacist-history-of-biological-sex/
InterACT: Advocates for Intersex Youth	InterACT: Intersex Brochures and Guides		[Internet]	https://interactadvocates.org/resources/intersex-brochures/
InterConnect	InterConnect: Resources		[Internet]	https://interconnect.support/what-we-do/
Intersex Justice Project	Intersex Justice Project: Intersex Activism Resources		[Internet]	https://www.intersexjusticeproject.org/resources.html
Lindahl H	Intersex person reacts to Middlesex	2020	YouTube [Internet]	https://www.youtube.com/watch?v=vmyKRGpZclc&list=PLlMq74isPDDb346zd9oq7A2WLem9tZKBk
Lindahl H	Writing an intersex character: 5 authenticity reader tips	2021	YouTube [Internet]	https://www.youtube.com/watch?v=w3VJsftZfcY&list=PLlMq74isPDDb346zd9oq7A2WLem9tZKBk&index=4
Lindahl H	Top tier intersex characters in fiction books (including Murakami)	2021	YouTube [Internet]	https://www.youtube.com/watch?v=_8Nwz0p_Ox4&list=PLlMq74isPDDb346zd9oq7A2WLem9tZKBk&index=5
Lindahl H, Brown K	XO, XXY, Oh My! Understanding Chromosomes and Prenatal Testing	2023 Jan 23	Hans Lindahl: Blog. [Internet]	https://hanslindahl.com/blog/sex-chromosomes-genetic-and-prenatal-testing
Scientific American	Medicine’s Fixation on the Sex Binary Harms Intersex People: A Question of Sex, Episode 2	2022 Aug 24	Scientific American video on YouTube [Internet]	https://www.youtube.com/watch?v=GFIugrTaSmM
Serano J	Transgender People and “Biological Sex” Myth	2017 Jul 17	Medium [Internet]	https://juliaserano.medium.com/transgender-people-and-biological-sex-myths-c2a9bcdb4f4a
Scamell D	The State of Intersex Funding: Funder Briefing	2019	Global Philanthropy Project [Internet]	https://s3.amazonaws.com/astraea.production/app/asset/uploads/2019/09/2019-Intersex-Funding-Brief.pdf

The resources listed here were compiled by the authors of this roundtable summary. This list focuses on biology education resources, biology research resources, biology perspectives on sex diversity and variation, interdisciplinary books on bodies, sex, and/or biology, and shorter-format intersex resources that are freely available online.

As addressed in the complementary article in this symposium collection, “Sex, Science, and Society: Reckonings and Responsibilities for Biologists,” the paradigm of binary sex needs a revolution. This paradigm is embedded in the institution of science: in scientific research, in science education, and in the norms and expectations of the production of scientific knowledge. We see this in the widespread conceptualization of sex diversity beyond female and male as an exception, a defect, an afterthought, or not even worth mentioning. These attitudes and approaches limit the research questions we can ask, the interpretation and further investigation of our findings, our capacity to inform through our teaching and science communication, and the inclusivity of our work and spaces. The ongoing erasure of intersex in our classrooms and curricula is not only a disservice to our students but furthers the medical, legal, and political harm committed against intersex communities, which is grounded in beliefs about the threat or impossibility of human sex trait variation. Here, symposium participants reflect on the binary sex paradigm and how science and culture co-create each other. Participants’ initials are used throughout to indicate specific reflections made during the symposium roundtable.

## Recognizing the diversity of sex and gender identities

Science has historically played, and continues to play, an unignorable role in the social, political, and medical oppression of queer, transgender, and intersex communities (Lewis and Sharpe 2023). Sex and gender are both complex and multifaceted, and they each need to be approached with much more nuance than academic biology typically offers. There is a remarkable level of diversity within and between sexes and sex traits of individuals across phyla, and this is typically framed as “other,” deviating from the binary, and deviant, rather than complex, multifaceted, and diverse (e.g., Rosario 2009; Roughgarden 2013). Ignoring the complexity of sex and gender does an incalculable disservice to a discipline that aims to understand the complexity of life on this planet.

The models that we are using to come up with questions about animal biology are thoroughly rooted in our cultural assumptions ([Bibr bib12][Bibr bib12]). As AEK noted in our roundtable discussion, “Cultural norms can be so pervasive that it’s almost as if they are ‘common sense,’ so it is hard for people who see them everywhere they look not to project them onto the biology that they’re studying.” Furthering this point, JFM added “When we are looking at systems like sexual selection with birds or lizards, and we are coming from a cultural background of ‘sex is binary,’ we’re asking questions like ‘why do we have different types of males that do different things’ or ‘why are these sex roles reversed between male and female.’ These are inherently rooted in the fact that we have a culture that looks at this as a binary, but birds and lizards don’t care!”

Experience and bias shape the questions asked by scientists, and in particular, by biologists. The dominant paradigm has been shaped by cisheteronormative culture, but this is rarely recognized as an actively subjective perspective. However, this is not the case for queer perspectives. For example, when queer, transgender, and intersex biologists push back against this paradigm, this is responded to as projecting our/their own perspectives onto non-human organisms. JFM continued, “I’ve had people be more pointed about it at me: ‘Well, you’re nonbinary, so you’re just projecting your own thing onto the animals,’ but if you’re cisgender and not interrogating the role of the binary in the questions you’re asking, you’re coming in with your own biases and your experience is shaping the questions you’re asking–it’s just that you don’t have to account for that in the same way as those of us who live outside those binaries do.”

Primate research is one place that has been particularly victim to projections of human cultural assumptions onto science (Haraway 1989; Kralick 2023). For example, there is a tendency to project the expectation that males will ideally be larger and stronger than females but her research on orangutan skeletons complicates this assumption, a topic that she has further explored in her publications (e.g., Kralick et al. 2023). A.E.K. notes that, while cultural norms are pervasive, queer experiences challenge what we are told are immutable, objective truths. Presenting the perspectives of queer people and diverse scientists can help everyone take such viewpoints into account and build approaches to biology that are more inclusive and equitable.

## Scientific communication requires inclusive language

As symposium participants and co-authors of various positionalities and perspectives, we collectively recognize the importance of centering the experiences and perspectives of queer, transgender, and intersex individuals in all discussions related to sex diversity in biology. The language used in biology classrooms, in scholarly scientific publications, and in public-facing scientific communication plays a role in the co-creation of science and culture (Subramaniam 2014). Language used in these spaces impacts oppressed communities and individuals; binary language that pathologizes sex and gender variation and diversity is actively harmful to queer, transgender, and intersex individuals both within and outside of science. H.L. emphasized that it is critical for biologists to be “aware of how language is used, and how that shapes knowledge production, and how that knowledge production is leaned upon by political and cultural actors” and underlined that intersex individuals “need allies, we need folks who are producing knowledge in a way that is sensitive to the social realities of our communities.”

Accompanying this roundtable summary, another article from this symposium surveys several terms in the literature that impose a binary framework onto sexual phenotypes (McLaughlin et al. 2023). These terms change in prevalence across time and among different taxa. One example is the conflation of sex and gender in non-human animals. Anthropomorphization is common in biology, in how organisms are presented in educational settings, and in how research is presented. A.A. explained “I’ve noticed a lot of the anthropomorphisms that you give are from a Western perspective. You end up trying to create these stories to make it familiar to your students and then you realize you’ve fed into some of the harmful cultural dynamics that can exist.” As such, anthropomorphization and ideas of human gender roles are particularly prevalent in ideas about “sex roles,” i.e., females provide care and males compete, typically slotting organisms into “female roles” and “male roles,” erasing the dynamic and unique behaviors, physiologies, and roles that diverse organisms embody. This mammal-centered framework of care vs. competition doesn’t accurately represent behavior or physiology across the animal kingdom; for example, in birds, biparental care is the norm.

Another example offered by S.L. is the discussion of the conflation of sex and gender in the terms “masculinize” and “feminize,” often implemented to refer to the effects of hormones such as testosterone, estrogen, and progesterone. She suggests alternative terms like “‘androgenize’ or ‘estrogenize’ could be more effective without conflating sex and gender. Alternatively, as biologists, we can talk about the levels of the hormones and the expression of the traits themselves, rather than framing these effects as belonging to one sex.” TJ also pointed out the limitations of anthropomorphization in the context of his research, “. . .fungi are so different than humans!. . . They don’t have to fit into ‘this is a male role’ or ‘this is like a female role,’ well, no, this is something unique that these organisms are doing.” Exploring such diverse forms of life without forcing analogies with human or mammalian behavioral roles and norms can help to destabilize ideas of a universal sex binary and ubiquitous normative gender roles.

As researchers and educators, we often fall into shorthands of lumping many things together as “sex.” While some use the term “sex” in reference to one trait such as chromosomes, gonads, gamete-production, or external genitalia, “sex” is often used to refer to many different traits with different distributions (Richardson 2022). JFM commented, “I found teaching that part of the way to work toward developing a new vocabulary for this is to be very specific about what you’re talking about.” An approach to minimize confusion, and to improve accuracy, is to *be specific*. Rather than calling a trait “sex-linked,” one can be specific about what a trait is linked to—a chromosome, and if so, which one. Rather than referring to “female” and “male” chromosomes, one can simply refer specifically to the chromosome(s) in question. As J.F.M. explained, this also offers additional benefits, “In general, taking the time to stop and ask ‘what trait or set of traits are we actually thinking about’ not only helps us break down these binaries, it also means we’re communicating better and more specifically—to both our colleagues in research, and when we’re teaching.”

A common term in biology is “sexual dimorphism,” which refers to a distinct or systematic difference between two sexes of a species. “Dimorphism” implies binary phenotypes, yet across populations two or more modes may exist, and those modes can have a range of relationships from overlapping to disjunct. Sex differences can also be fluid or dynamic over time, as sex phenotypes may shift during the natural aging process or even fully interconvert as in sequential hermaphroditism. Given this range of variation, there may be a need to consider different terminology. Many alternatives have been proposed, such as sex differences, sexual difference, sex variation, sex polymorphism, and sexual heteromorphism (reviewed in Kralick et al. 2023). A.A. shared that he uses “sexual heteromorphism,” for example, because “I think that’s more inclusive because it conveys there’s that wider range, and it also makes room for those multimodal traits” (Anderson and Falk 2023; Anderson and Renn 2023). Even in systems where dioecious gamete production has been historically considered a fundamental binary, this allows for the description of a wider range of sex morphs, and distributional differences between gamete type and other traits. However, the term “sexual dimorphism” has been used by researchers for a long time and many understand the way the term fails to reflect the diversity of sex traits and sex expression, but are unsure how to incorporate a different term. A.E.K. explained “Something we can do as a community beyond just suggesting new terminology, is to model how to use alternative terminology in our own science, call people in for these discussions rather than call them out, and create space for them to work through how this makes sense for them and in the work that they’re doing.”

## The role of scientific institutions in addressing systemic biases

It’s important for scientific institutions to be aware of the biases and assumptions that are embedded within their practices and to actively work to address them.

We need to ask ourselves tough questions about the structures of power that exist within the scientific community and how those structures perpetuate inequality and oppression. Academic science departments are influential sites of knowledge production and dissemination within a hegemonic paradigm that presents itself as ahistorical and value neutral. BS commented, “Biologists are trained and will usually teach only the details of biology—they do not teach the history of biology or where ideas come from.” As a result, the embedded assumption that a fundamental and organic sex binary exists in nature continues to be passed on from instructors to students without attentiveness to the cultural and political genesis of this paradigm. When biology curricula only discuss sex as a binary, gender as a culturally mediated extension of sex, and sexuality as always heterosexual and reproductively oriented, this reinforces the implicit belief that these are the only normal, natural, and biologically codified ways for organisms to exist. I.C. points out “This has another layer of importance because of the political implications for this, as well, when it’s transferred to humans, which we do so readily.” The absence of queerness and sex variation in research and pedagogy within academic biology furthers political arguments that these characteristics are unnatural and in need of eradication.

## The importance of including diverse perspectives in scientific research

Science and culture co-create each other (Subramaniam 2014). Contemporary approaches to and experiences of intersex individuals in the US have been unquestionably shaped by European colonialism. A.R.W. commented “The fact that there’s no intersex representation in culture, period—I’m saying ‘no’ hyperbolically but very little, empirically—it reinforces the idea that we’re not there, that we don’t exist.” A common narrative of intersex people is discovering being intersex as a teenager or adult after learning about intersex in a magazine, in a book, online, or in class. Likewise, intersex people have little-to-no visibility in science and medicine, and scholarly articles, especially scientific scholarly articles, typically exclude intersex individuals and related content.

Intersex traits generally appear in the scientific literature only as a source of isolated pathology. While some intersex traits do present with accompanying health problems, sex variation itself is frequently pathologized as a source of aesthetic and social disruption requiring medical treatment. As a result, intersex populations are excluded from general health research, resulting in both a failure to accurately account for the spectrum of human diversity in such research and preventing appropriate care protocols from being developed to accommodate intersex health needs. There is an increasing recognition that the positionality and perspectives of those producing science have tremendous influence on scientific knowledge production and on “the science” as we think of it.

## The impact of scientific research on policy and public perception

As symposium roundtable participants and co-authors, we advocate for the inclusion of queer, transgender, and intersex perspectives and experiences in *all* levels of science education and research.

Science cannot afford to be ignorant to LGBTQIA+ contributions, including related to gender and sex. We must be aware of how language is used, by ourselves, by our colleagues, and by those outside of academic science. Our language shapes knowledge production, and that knowledge production is leaned upon by political and cultural actors. HL commented “I’d love to see scientists and knowledge makers organizing with each other to do things like get in the media and denounce appeals to ‘science’ and ‘biology’ in denying intersex and transgender people healthcare and civil rights.” Legislators writing bills often demand data. For example, in bills that proposed to delay medically unnecessary surgeries on intersex babies and children, legislators requested data to support the proposed bills. H.L. recommended that scientists follow the lead of intersex leaders and activists by “importing those sorts of language ideas. . ., working alongside community organizations like InterACT, Intersex Justice Project, The Houston Intersex Society, and other folks who are collaborating with researchers on these topics and of course just speaking up for these ideas, challenging these paradigms.” The paradigm of binary sex must be challenged by scientists working *with* those who are queer, trans, and intersex and creating opportunities for members of these communities to prevail in STEM fields themselves as well.

## The need for education and outreach efforts

Academic biologists whose bodies and lives are not consistently called into question by the heteronormative and binary paradigms recapitulated in disciplinary pedagogy may be unaware of their pervasiveness. While collective engagement is necessary for structural transformation, providing clearly defined additions or modifications to standard lessons can make it easier for a broader swath of instructors to begin creating change in their classrooms. Sharing resources and language swaps can help assuage concerns that substantial expertise or perfected fluency are necessary for bringing about a more queer, trans, and intersex inclusive perspective to biology classes. Several members of our roundtable offered specific texts (see Table 1), strategies, and learning objectives toward these ends.

A.K.L.: In my 100-level interdisciplinary gender, biology, and health course, I use Viloria and Nieto’s book *The Spectrum of Sex: The Science of Male, Female, and Intersex* (2020) to introduce sex during the first week of the semester. The first discussion about sex, gender, and bodies centers Hida Viloria’s experience as an intersex person. In this same class period, we discuss terminology, and I tell them why I use specific, accurate terms, rather than gendered or sexed terms to refer to anatomical or physiological traits. When I teach about the development of sex traits, we talk about how variable sex traits can be at any given moment and throughout a lifetime. I refer to the dominant paradigm as the binary sex paradigm and to *this work* as part of the sex-diverse paradigm. We also explore feminist psychology and feminist neuroscience where there are lively (and related) debates about binary sex and gender essentialism. Later in the semester, students read two of Cary Gabriel Costello’s Intersex Roadshow blogposts (Costello 2011a, b) and explore the InterACT website (InterACT: advocates for Intersex Youth) and read their FAQs and brochures.

S.L.: In my 200-level genetics course, I talk about X- and Y-linked genes, rather than “sex-linked” genes. This turns out to be quite useful when constructing Punnett squares and interpreting pedigrees because it helps students clue into chromosomal patterns of inheritance. I’ve learned a lot from Jess’s blog on this topic (McLaughlin 2022).

S.S.: When I've taught and TA'ed biology courses with a more prescribed curriculum and limited time for extra content, I take part of one class to talk about the relationship between dioecy and monoecy, sex, and gametes. I explain how these terms will be used in the course and note that this doesn’t encompass the full complexity of sex and is not interchangeable with gender. I also invite students to contact me to discuss this topic further if they are interested. In the Gender Studies course I’ve taught, I spend more time talking about human biological sex trait variation and the specific social history that has led to the construction of a sex binary. I assign them a short essay that provides a summary of the history of the sex and gender binary (Henderson 2020) and a TEDTalk from Emily Quinn (Quinn 2019) which helps humanize intersex experiences to students who are not familiar with intersex.

## The importance of interdisciplinary collaboration

The life experiences and biological realities of queer, trans, and intersex people are frequently treated as strictly cultural phenomena or broadly erased (in the case of intersex individuals), precluding their consideration by academic biologists. However, in order to bring about recognition of and freedom from oppression for these populations, invested collaborations between biologists, humanities scholars, and community members are critical. As BS pointed out, the failure to consider LGBTQIA+individuals as equally natural, normal, and biological within cultures shaped by European colonial influence “is possible because academia is filled with disciplinary silos. I think that the more interdisciplinary we can be in historicizing what we do in our classrooms and in our research, students will understand how scientific knowledge is produced, and it will go a long way toward breaking some of these assumptions.” H.L. echoed a similar sentiment, “There is so much diversity of human thought, but science exists within culture, so what are you going to do besides work toward shifting culture? They work together in tandem.”

## Funding

Funding for the Society-Wide Symposium on "Sex Diversity and Variation" at the 2023 Society for Integrative and Comparative Biology Annual Meeting during which the Roundtable Discussion took place was provided by the Society for Integrative and Comparative Biology.

## Authors’ positionality statement

While scientific research and teaching are often presented as objective, value-neutral, and devoid of social context, science is constitutively shaped by social, cultural, political, and historical factors. Our work interrogates these dynamics and intentionally includes perspectives from populations historically harmed and excluded through biomedical and evolutionary research on sex and sex diversity. This paper was co-authored by a group that includes both academics and activists, several of whom are members of the LGBTQIA+community. As such, we recognize that the dialogue of the roundtable and these resulting recommendations have been informed by our various experiences, perspectives, backgrounds, and identities. We draw on intersex activist work, as well as the fields of feminist science studies, gender studies, biology education, and comparative biology in our dialogue and recommendations.

## Notes

From the symposium “Sexual diversity and variation” presented at the annual meeting of the Society for Integrative and Comparative Biology, January 4, 2023.
